# Intestinal parasites co-infection and associated factors among active pulmonary tuberculosis patients in selected health centers, Addis Ababa, Ethiopia: unmatched case control study

**DOI:** 10.1186/s12879-019-4009-0

**Published:** 2019-05-10

**Authors:** Ayinalem Alemu, Abebaw Kebede, Biniyam Dagne, Misikir Amare, Getu Diriba, Bazezew Yenew, Ephrem Tesfaye, Mengistu Tadesse, Waganeh Sinshaw, Dawit Challa, Kassu Desta

**Affiliations:** 1 Ethiopian Public Health Institute, Addis Ababa, Ethiopia; 2 Department of Medical Laboratory Science, College of Health Sciences, Addis Ababa University, Addis Ababa, Ethiopia

**Keywords:** Pulmonary tuberculosis, Intestinal parasites, Co-infection, Associated factors

## Abstract

**Background:**

In co-endemic areas, rate of intestinal parasites and tuberculosis (TB) co-infection thought to be high. However, there are limited studies on the epidemiology of this co-infection in Ethiopia. Therefore, the present study aimed to generate evidence on intestinal parasites co-infection rate and associated factors among pulmonary tuberculosis patients (PTB) and their household contacts in Addis Ababa, Ethiopia.

**Methods:**

Unmatched case-control study was conducted. Data were collected from 91 PTB patients (cases) and 89 household contacts (controls). Socio-demographic characteristics and associated factors were collected using structured questionnaire. Sputum, stool and blood specimens were collected, processed and examined for PTB, intestinal parasites and *Human Immunodeficiency virus* anti-body test, respectively. Data were entered and analyzed by Statistical Packages for Social Sciences (SPSS) Version 20. Descriptive statistics, Fisher’s exact test, binary logistic regression, and odds ratio were used. *P-value* of < 0.05 was considered as statistically significant.

**Results:**

The infection rate of intestinal parasites based on one stool samples in PTB patients and controls was 22 and 9%, respectively. The difference was statistically significant (COR = 2.85;95% CI = 1.18–6.87). The most prevalent intestinal parasite in PTB patients was *Gardia lamblia* (8.8%, 8), followed equally by *Ascaris lumbricoides*, *Haymenolopsis nana* and *Entamoeba histolytica/dispar* (4.4%, 4). Co-infection in PTB patients was associated with body mass index (BMI) < 18.5 (AOR = 6.71;95% CI = 1.65–27.25) and dirty material in finger nails (AOR = 8.99;95% CI = 2.46–32.78). There was no variable associated with parasitic infections in controls in our analysis, which might be due to the low prevalence of intestinal parasites’.

**Conclusions:**

There was a statistical significant difference in the infection rate of intestinal parasites in PTB patients compared to healthy household contacts. The consequence of co-infection on developing an active disease, disease severity and treatment efficacy needs to be investigated in future.

## Background

Tuberculosis is the leading cause of death from a single infectious agent globally [1]. The problem is worse in high burden countries [1]. The 30 high TB, multi drug resistance-TB and TB/ *Human Immunodeficiency virus* (HIV) burden Countries accounted 90% of all estimated incident cases worldwide [1]. Although one third of the world population is estimated to be infected with *Mycobacterium tuberculosis* (*MTB*)*,* relatively a small proportion develops TB [1–3]. The host’s immune system is a key factor for the progression of *MTB* infection [4, 5]. Susceptibility to the disease is associated with reduced Th1 type responses [3]. It was reported that intestinal parasites (IP), specifically helminthes causes immune activation with biased T helper 2 responses and down regulated Th1 and Cytolytic T lymphocytes activity [6]. This change in the immunological milieu of the host might impair the immunological response to pathogens which need Th1 responses to limit the severity and progression of infection [6]. In sub-Saharan Africa, where the prevalence of parasitic infections is very high, a dominant Th2 polarized immune response has been reported and suggested to increase susceptibility to *MTB* [3, 7].

It is estimated that nearly 3.5 billion people are affected, and 450 million are ill due to parasite (protozoa or helminthes) infections globally [5, 8]. TB and IPs affect primarily low social and economic level populations [5]. Both infections overlap substantially in geographic distribution [3, 9]. Thus, the likely hood of co-infection is thought to be high. Epidemiology of this co-infection was reported in different countries [3, 5, 7, 9, 11–15]. Both infections are the major public health problems at national and regional level in Ethiopia [6, 11]. Higher prevalence of IPs co-infection among TB patients was reported from Gondar, Ethiopia [3]. In a recent study from Arbaminch, Ethiopia a 26.3% of intestinal helminthes co-infection rate was reported [12]. This co-infection would increase the complexity of control and prevention of TB and parasitic diseases [9–16]. It is described that Bacille Calmette Guerin (BCG) has a limited effect against TB epidemic in developing countries where the prevalence of intestinal helmenthiasis is high [16–18].

Understanding co-infection rate has an input to design effective mechanism to reduce mortality due to dual effect and for designing effective prevention and control mechanisms. However, there are limited studies in the country which are concentrated in similar settings. Therefore, the present study aimed to assess intestinal parasite co-infection rate and associated factors among active PTB patients in highly populous Sub-city of Addis Ababa (urban setting), Ethiopia.

## Methods

### Study setting and design

Unmatched case-control study was conducted during the period between Jan 2017 to Jan 2018, in selected health centers of *Kolfe Keraniyo* Sub-city*,* Addis Ababa, Ethiopia. Addis Ababa is the capital city of Ethiopia, with an altitude ranging from 2100 m to 3000 m above sea level [19]*.* Among the ten Sub-cities in the city, *Kolfe Keraniyo* Sub-city was selected purposely due to easier access to health centers and patient flow. Three health centers which provide care and treatment for TB and HIV/AIDS patients were randomly selected, namely; *Kolfe, Wereda 11* and *Lomi meda* health centers.

### Eligibility criteria and sampling

All PTB patients visiting TB clinics and their household contacts were used as study subjects. Cases were individuals with bacteriological confirmed active PTB such that all individuals with smear positive results were included. For confirmation Culture and Xpert MTB/RIF Assay were performed for all cases and included after confirmation. Controls were healthy household contacts to active PTB patients with no clinically and bacteriological diagnosed TB. All age groups who fullfilled the definition of cases/controls, volunteered to take part and gave written informed consent were included in the study. However, *HIV* positive individuals and TB patients on treatment excluded from the study. All controls were screened for TB symptoms and PTB was ruled out by using a combination of bacteriological diagnostic methods; Smear microscopy, Culture and Xpert MTB/RIF Assay. Sample size of 178 (89 cases, 89 controls) was obtained by using OpenEpi, version 3 sample size calculation for unmatched case control study by Fleiss with continuity correction (prevalence in cases; 50, prevalence in controls; 28.5, α; 0.05, power; 80, ratio; 1:1), based on an average of two studies conducted at Gondar, Ethiopia [3, 7]. Nonrandom convenience sampling technique was used; such that consecutive cases and controls were enrolled. Written informed consent was administered for volunteers after full explanation of the study. Socio-demographic characteristics and associated factors were collected using a pretested structured questionnaire via face to face interview by trained nurses. BCG vaccination status was collected by nurses by looking BCG scar on participants’ arms. Shoe wearing was defined as a habit of frequent shoe wearing any where being outside home. Similarly presence of any foreign body in the participant’s finger nail (untrimmed) was considered as presence of dirty material. Laboratory examinations including sputum smear microscopy, Xpert MTB/RIF assay, sputum culture, stool examination and HIV antibody test were done by senior laboratory technologists.

### Laboratory diagnosis

Two spot sputum specimens for smear microscopy and one morning sputum specimen volume of 5 ml for Xpert MTB/RIF assay and culture were collected based on Ethiopian TB and leprosy control guideline [20]. Smear microscopy was done by using auramine O staining method and examined by fluorescent microscope [21]. Sputum samples for culture and Xpert MTB/RIF assay were stored at 2-8^o^c until transported to Ethiopian Public Health Institute, National Tuberculosis Reference Laboratory. Samples were processed by N-Acetyl-L-Cysteine–Sodium Hydroxide (NALC-NaOH) decontamination method as described by GLI mycobacteriology laboratory manual [22]. Phosphate buffere (pH 6.8) solution (PBS) was used to for homogenization and re suspension. Re-suspended sediment was inoculated in to both solid (LJ) and liquid (MGIT) medias. Prior to inoculation, 800 μl from a mix of 15 ml MGIT growth supplement and PANTA was added to MGIT tubes [22]. LJ slants were incubated at 37 °C, while MGIT tubes were loaded to MGIT 960 instrument (Becton-Dickinson and Company, Sparks MD). LJ slants not had colonies growing at eighth weeks and no growth unit on MGIT tubes at 42 days were defined as negative [22, 23]. SD BIOLINE TB Ag MPT 64®(STANDARD DIAGNOSTICS, INC, Republic of Korea) test was used for the confirmation of *Mycobacterium tuberculosis* complex [24]. For Xpert MTB/RIF assay 0.5 ml of sediment was mixed with 1.5 ml of sample reagent buffer (supplied in kit). After 15 min, an approximate of 2 ml of the specimen was dispensed it into Xpert MTB/RIF’s cartridge and loaded to GeneXpert instrument (Cepheid, Sunnyvale, CA, USA). The result was viewed after 2 h [25].

An approximate of a three gram of stool sample was collected and processed for parasitic examination using Wet mount, Formol-ether concentration and Modified ZN method. For wet mount**;** an approximate of 50 mg of feces mixed with a drop of normal saline on a clean slide and screened systematically for the presence of helminth ova and larvae or protozoan cysts and trophozoites [26]. For formol-ether concentration, about 1 g of faeces was placed in a clean 15 ml conical centrifuge tube containing 7 ml formalin saline. The suspension was filtered through a sieve into another conical centrifuge tube and 3 ml of diethyl ether was added. The contents were centrifuged at 3, 200 rpm for 3 min and the supernatant was poured away. Two smears were made on clean glass slides and the entire area of one smear was systematically examined by using 10x and 40x objective lenses [11]. From the other smear, modified ZN staining method was used to stain coccidians. After fixing the smear with methanol; 1% carbol fuchsin, 1% v/v acid alcohol and 0.25% malachite green were used as a primary stain, decolorizer and counter stain respectively. The smear was examined by experienced microbiologist using 100x objective [27].

HIV antibody test on TB patients was done by using rapid test kits (Wanti HIV1 + 2, Uni-Gold™ HIV and VIKIA HIV 1/2 [28]. Interpretation was based on the combination of these three rapid tests such as Wanti HIV1 + 2 as a screening test, Uni-Gold™ HIV as a confirmatory test and Uni-Gold™ HIV and VIKIA HIV 1/2 was used as a tie breaker.

### Data quality control

A structured questionnaire was prepared and pre-tested on patients who did not include in the study. Nurses working in TB clinics and laboratory technologists of each health center were selected and trained. The collected data were checked for completeness, accuracy, clarity,and consistency by the principal investigator, on daily basis. Controls were run and preventive maintenance was performed to ensure the quality of instrument performance. Reagents were checked for reliability and reproducibility of the test before any test started. Appropriate sputum and stool specimens were collected, processed and examined according to standard operating procedures. For all smear positive PTB patients, Xpert MTB/RIF and sputum culture were performed for confirmation. Stool examination was conducted by individuals who were blinded to the source of the specimen whether it was from TB patients or from controls. Control slides were used. The result of laboratory examination was recorded on well-prepared format carefully. Prepared LJ media was checked visually for the color, texture,and homogeneity, and performance check and sterility test were performed before usage. For Liquid culture, everything was done based on MGIT instrument manual [29].

### Statistical analysis

Data were coded, checked, entered, stored andanalyzed by SPSS Ver. 20. Q-Q plot was used to check normal distribution for age, BMI and monthly income. Descriptive statistics such as frequencies, proportions, mean and standard deviation were used to explain socio-demographic, associated factors and intestinal parasites infection rate. The association between intestinal parasites and TB co-infection was evaluated by using binary logistic regression. To analyze associated factors for the co-infection univariate analysis (binary logistic regression) was done. For all variables with a *P-value* of ≤0.28 in the univariate analysis, multivarate analysis was done sequentially by removing the variable with largest *P-value* until statistically significant associations were found. Fisher’s exact test was used to compare the study variables for the presence or absence of association for variables not fulfilled the assumptions of Chi-squared test. Odds ratio and 95% confidence interval was used to measure the strength of an association. *P*-value < 0.05 was considered indicative of a statistically significant difference.

## Results

### Socio-demographic characteristics

**Fig. 1:**
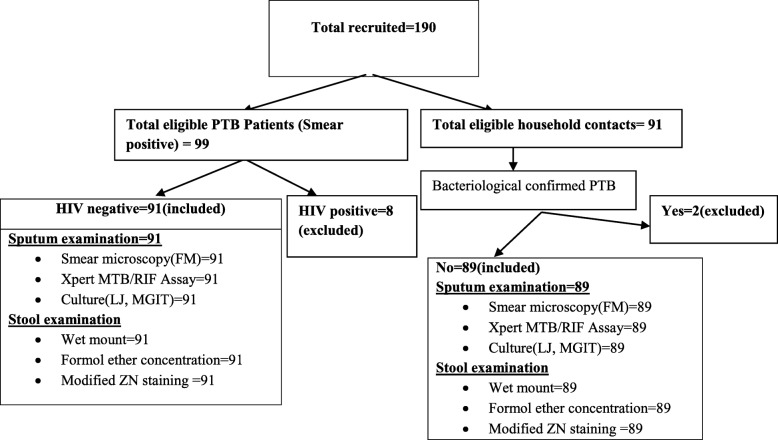
Flow chart for enrollment and data collection procedure for pulmonary tuberculosis patients and household contacts in selected health centers of *Kolfe Keraniyo* Sub-city, Addis Ababa, Ethiopia, Jan 2017 to Jan 2018

A total of 190 study participants were enrolled in this study; of them 99 were cases with bacteriological confirmed active PTB and 91 were controls that were healthy household contacts to TB patients. Eight from the cases and two from the controls were excluded due to HIV co-infection and bacteriologically confirmed PTB respectively. Therefore, 91 cases and 89 controls were included in the analysis (Fig. 1). Majority (61.5%) among the cases were males, while females accounted largest proportions (62.9%) among the controls. The mean age of cases and controls was 26.7(±7.7) and 26.69 (±9.19) years, respectively. More than 90% of cases and controls were younger than 40 years. The residence for 97.8% of both cases and controls were from urban. Around 66% from the cases and the controls were unemployed. Both cases and controls had been utilizing tap water as a source of drinking water equally (97.8%). Eighty five (93.4%) from cases and all of the controls reported that they had latrine or use latrine. All cases and majority of the controls (98.9%) had a shoe wearing habit. Similarly all from both groups have been washing their hands before taking meal, at breakfast, lunch and dinner time. A habit of hand washing practice after toilet was reported by 82.4% of cases and 85% of controls. However, 40% of cases and 41.2% of controls were washing their hands by using water without any soap or detergent. In 21(23.1%) cases and 13(14.6%) controls there was a dirty material in their finger nails (Table 1). The mean body Mass Index (BMI) of PTB patients was 18.26 (±2.14). Among PTB patients 17.6% had a scar on their arm for BCG vaccination. Among house hold contacts, 19.1% had a scar.

**Table 1:** Socio-demographic and behavioral characteristics of tuberculosis patients (*n* = 91) and controls (*n* = 89) in selected health centers of *Kolfe Keraniyo* Sub-city, Addis Ababa, Ethiopia, Jan 2017 to Jan 2018

	Variables	Cases Number (%)	Controls Number (%)
Age	Mean	26.7	26.96
	SD	7.69	9.19
Age group	≤24	44(48.4)	38(42.7)
	25–34	31(34.1)	34(38.2)
	≥35	16(17.6)	17(19.1)
	Total	91(100.0)	89(100.0)
Sex	Male	56(61.5)	33(37.1)
	Female	35(38.5)	56(62.9)
	Total	91(100.0)	89(100.0)
Residence	Urban	89(97.8)	87(97.8)
	Rural	2(2.2)	2(2.2)
	Total	91(100.0)	89(100.0)
Marital status	Single	56(61.5)	43(48.3)
	Married	35(38.5)	46(51.7)
	Total	91(100.00)	89(100.0)
Educational status	No formal education	26(28.6)	27(30.3)
	Primary completed	42(46.2)	25(28.1)
	High school completed	19(20.9)	20(22.5)
	College &above	4(4.4)	17(19.1)
	Total	91(100.0)	89(100.0)
Monthly income	Low	46(50.5)	28(31.5)
	Medium	22(24.2)	25(28.1)
	Satisfactory	23(25.3)	36(40.5)
	Total	91(100.0)	89(100.0)
Latrine availability	Yes	85(93.4)	89(100.0)
	No	6(6.6)	0(0.0)
	Total	91(100.0)	89(100.0)
Raised poultry	Yes	8(8.8)	22(24.7)
	No	83(91.2)	67(75.3)
	Total	91(100.0)	89(100.0)
Occupation	Employed	31(34.1)	30(33.7)
	Unemployed	60(65.9)	59(66.3)
	Total	91(100.0)	89(100.0)
Swimming habit	Yes	6(6.6)	3(3.4)
	No	85(93.4)	86(96.6)
	Total	91(100.0)	89(100.0)
Habit of shoe wearing	Yes	91(100.0)	88(98.9)
	No	0(0,0)	1(1.1)
	Total	91(100.0)	89(100.0)
Bathing	Home	87(95.6)	87(97.8)
	River	4(4.4)	1(1.1)
	Home and River	0(0.0)	1(1.1)
	Total	91(100.0)	89(100.0)
Hand wash before meal	Yes	91(100.0)	89(100.0)
	No	0(0.0)	0(0.0)
	Total	91(100.0)	89(100.0)
Water source for drink	Tap	89(97.8)	87(97.8)
	River	0(0.0)	0(0.0)
	Tap and River	2(2.2)	2(2.2)
	Total	91(100.0)	89(100.0)
Washing cloth	Home	85(93.4)	88(98.9)
	River	2(2.2)	0(0.0)
	Home and River	4(4.4)	1(1.1)
	Total	91(100.0)	89(100.0)
Dirty material in the finger	Yes	21(23.1)	13(14.6)
	No	70(76.9)	76(85.4)
	Total	91(100.0)	89(100.0)
Eating unwashed vegetables	Yes	28(30.8)	19(21.3)
	No	63(69.2)	70(78.7)
	Total	91(100.0)	89(100.0)
Eating raw meat	Yes	52(57.1)	38(42.7)
	No	39(42.9)	51(57.3)
	Total	91(100.0)	89(100.0)

### The burden of intestinal parasites

The overall infection rate of intestinal parasites among PTB patients and controls was 22%(20/91) and 9% (8/89) respectively. The difference was statistically significant (COR = 2.85; 95% CI = 1.18–6.87). A total of 24 intestinal parasites (14 intestinal protozoans and 10 intestinal helminthes) from PTB patients and eight intestinal parasites (six intestinal protozoans and two intestinal helminthes) from controls were identified. There was a statistical significance difference for helminthic infection between PTB patients and controls (COR = 5.37; 95% CI = 1.14, 25.25), but not for protozoans(*p = 0.157*). Multiple parasitic infections were found in four (4.4%) PTB patients; but not in controls (*p = 0.045*). Among all intestinal parasites identified in PTB patients; *G.lamblia* was frequently found (8, 33.3%), followed by the equal prevalence of *A. lumbricoides, H.nana and E.histolytica/dispar* (4, 16.7%). *E.histolytica/dispar* was frequently identified from controls (5, 62.5%). Among all intestinal parasites identified *G.lamblia*, *A.lumbricoides* and *H.nana* were found with a statistical significance difference (*p = 0.046*, *p = 0.045* and *p = 0.045* respectively) between cases and controls (Table 2).

**Table 2:** Infection rate of intestinal parasites among tuberculosis patients (*n* = 91) and controls (*n* = 89) in selected health centers of *Kolfe Keraniyo* Sub-city, Addis Ababa, Ethiopia, Jan 2017 to Jan 2018

Variables	Cases Number (%)	Controls Number (%)	COR(95% CI)
Parasites			
No	71 (78.0)	81(91.0)	1.00
Yes	20 (22.0)	8(9.0)	2.85(1.18–6.87)
Total	91(100.0)	89(100.0)	
Protozoa			1.00
No	79(86.8)	83(93.3)	2.10(0.75–5.87)
Yes	12(13.2)	6(6.7)	
Total	91(100.0)	89(100.0)	
Helminthes			
No	81(89.0)	87(97.75)	1.00
Yes	10(11.0)	2(2.25)	5.37 (1.14–25.25)
Total	91(100.0)	89(100.0)	
≥2 parasites			–
No	87(95.6)	89(100.0)	
Yes	4(4.4)	0(0.0)	
Total	91(100.0)	89(100.0)	
Each parasite			
*E.histolytca/dispar*	4(16.7)	5(62.5)	
*G.lamblia*	8(33.3)	1(12.5)	8.48 (1.04–69.29)
*C. parvum*	2(8.3)	0(0.0)	
*A.lumbricoids*	4(16.7)	0(0.0)	–
*S.sterocolaris*	0(0.0)	1(12.5)	
*Taenia spp*	0(0.0)	1(12.5)	
*H.nana*	4(16.7)	0(0.0)	–
*T.trichuria*	2(8.3)	0(0.0)	
Total	24(100.0)	8(100.0)	

*COR* Crude Odds ratio, *CI* Confidence Interval,“ -” = Not Done

### Associated factors for intestinal parasites infection

Intestinal parasitic co-infection among PTB patients had a statistically significant association BMI (AOR = 6.71;95% CI = 1.65–27.25) and presence of dirty material in the participant’s finger (AOR = 8.99;95% CI = 2.46–32.78) (Table 3). However; Intestinal parasitic infection in the control group had not statistically significant association with any of socio-demographic or behavioral factors (Table 4, Table 5). Intestinal parasites are identified from 6(37.5%) of BCG vaccinated PTB patients and the majority (4, 66.7%) were intestinal helminthes.

**Table 3:** Associated factors for intestinal parasites co-infection among tuberculosis patients (*n* = 91) in selected health centers of *Kolfe Keraniyo* Sub-city, Addis Ababa, Ethiopia, Jan 2017 to Jan 2018

Variables	Cases					
	Stool examination result		COR(95% CI)	*p-value*	AOR(95% CI)	*p-value*
	Parasites detected, N*(%)	Parasites not detected, N*(%)				
Gender				0.873	–	–
Male	12(60.0)	44(62.0)	1.00			
Female	8(40.0)	27(38.0)	1.08(0.39–2.99)			
Age group				0.700	–	–
10–24	8(40.0)	36(50.7)	0.66(0.17–2.16)			
25–34	8(40.0)	23(32.4)	1.04(0.26–4.18)			
≥ 35	4(20.0)	12(16.9)	1.00			
BMI				0.025		0.008
< 18.49	16(80.0)	36(50.7)	3.88(1.18–12.78)		6.71(1.65–27.25)	
18.5–24.99	4(20.0)	35(49.3)	1.00		1.00	
Marital status				0.381	–	
Single	14(70.0)	42(59.2)	1.00			
Married	6(30.0)	29(40.8)	0.62(0.21–1.80)			
Educational status				0.329	–	–
No formal education	4(20.0)	22(31.0)	1.00			
Primary completed	8(40.0)	34(47.9)	1.29(0.34–4.81)			
Completed high school	6(30.0)	13(18.3)	2.53(0.60–10.70)			
Collage & above	2(10.0)	2(2.8)	5.50(0.59–51.19)			
Occupation				0.141		0.366
Employed	4(20.0)	27(38.0)	1.00		1.00	
Unemployed	16(80.0)	44(62.0)	2.45(0.74–8.11)		1.86(0.48–7.22)	
Monthly income				0.280		0.186
Low	12(60.0)	34(47.9)	1.00(0.32–3.12)		1.46(0.34–6.14)	
Medium	2(10.0)	20(28.1)	0.28(0.05–1.59)		0.23(0.03–1.73)	
Satisfactory	6(30.0)	17(24.0)	1.00			
Dirty material in the finger				0.002		0.001
Yes	10(50.0)	11(15.5)	5.45(1.83–16.17)		8.99(2.46–32.78)	
No	10(50.0)	60(84.5)	1.00		1.00	
Eating unwashed vegetables				0.933	–	
Yes	14(70.0)	22(31.0)	0.95(0.32–2.81)			
No	6(30.0)	49(69.0)	1.00			
Eating raw meet				0.466	–	–
Yes	10(50.0)	22(31.0)	0.69(0.25–1.87)			
No	10(50.0)	49(69.0)	1.00			
Raised poultry/livestock				0.829	–	–
Yes	2(10.0)	6(8.5)	1.20(0.22–6.48)			
No	18(90.0)	65(91.5)	1.00			

*COR* Crude odds Ratio, *AOR* Adjusted Odds Ratio, *CI* Confidence Interval,“ -” Not Done, N* Number

**Table 4:** Associated factors for intestinal parasites co-infection among healthy household contacts (*n* = 89) in selected health centers of *Kolfe Keraniyo* Sub-city, Addis Ababa, Ethiopia, Jan 2017 to Jan 2018

Variables	Controls			
	Stool examination result		COR(95% CI)	*p-value*
	Parasites detected, N*(%)	Parasites not detected, N*(%)		
Gender				0.979
Male	3(37.5)	30(37.0)	1.02(0.22–4.57)	
Female	5(62.5)	51(63.0)	1.00	
Educational status				0.387
1^o^ completed or less	4(50.0)	48(59.2)	0.38(0.07–1.94)	
Completed high school	1(12.5)	19(23.5)	0.24(0.02–2.61)	
College & above	3(37.5)	14(17.3)	1.00	
Occupation				0.315
Employed	4(50.0)	26(32.1)	1.00	
Unemployed	4(50.0)	55(67.9)	0.47(0.11–2.04)	
Dirty material in the finger				0.392
Yes	2(25.0)	11(15.6)	2.12(0.37–11.86)	
No	6(75.0)	70(86.4)	1.00	
Eating unwashed vegetables				0.254
Yes	3(37.5)	16(19.8)	2.43(0.52–11.28)	
No	5(62.5)	65(80.2)	1.00	
Eating raw meat				0.247
Yes	5(62.5)	33(40.7)	2.42(0.54–10.84)	
No	3(37.5)	48(59.3)	1.00	
Raised poultry/livestock				0.415
Yes	1(12.5)	21(25.9)	0.40(0.04–3.51)	
No	7(87.5)	60(74.1)	1.00	

*COR* Crudes Odds Ratio, *CI* Confidence Interval, N*(%) = Number

**Table 5:** Associated factors for intestinal parasites co-infection among tuberculosis patients (*n* = 91) and controls (*n* = 89) in selected health centers of *Kolfe Keraniyo* Sub-city, Addis Ababa, Ethiopia, Jan 2017 to Jan 2018

Variables	Cases			Controls		
	Number (%)	Fisher’s exact test	*p-value*	Number (%)	Fisher’s exact test	*p-value*
Residence		7.26	0.046		0.00	1.000
Urban	18(90.0)			8(100.0)		
Rural	2(10.0)			0(100.0)		
Latrine availability		0.69	0.44		–	–
Yes	20(100.0)			8(100.0)		
No	0(0.0)			0(0.0)		
Swimming habit		0.69	0.404		0.22	0.636
Yes	0(0.0)			1(12.5)		
No	20(100.0)			7(87.5)		
Bathing		0.21	0.640		6.33	0.173
Home	20(100.0)			7(87.5)		
River	0(0.0)			0(0.0)		
Home and River	0(0.0)			1(12.5)		
Hand wash style after toilet		1.13	0.286		14.08	0.495
With water	10(50.0)			2(25.0)		
With water &soap	10(50.0)			6(75.0)		
Water source for drink		0.00	1.000		0.00	1.000
Tap	20(100.0)			8(100.0)		
River	0(0.0)			0(0.0)		
Tap and River	0(0.0)			0(0.0)		
Washing cloth		0.91	0.740		0.00	1.000
Home	20(100.0)			8(100.0)		
River	0(0.0)			0(0.0)		
Home &River	0(0.0)			0(0.0)		

“-” = Not Done

## Discussion

TB and parasitic diseases overlap similar geographic distribution, especially in developing countries [7, 16]. The overall intestinal parasite co-infection rate among active PTB patients in this study was 22%. This is similar with studies done at Arbaminch, Ethiopia [12] and Brazil [5]. However, it is lower than other studies in Gondar, Ethiopia [3, 13] and Brazil [14]. Three consecutive stool specimens examined in these studies might be a reason for the lower results in the present study. These studies were conducted 15 years ago which might be another possible reason, because currently health extension workers are engaged in the primary health care activities across the nation. Inaddition, co-infection was lower than other studies conducted in Gondar, Ethiopia [7, 11] and in Tanzania [15]. The difference in the study area, where the present study was conducted in urban setting might be a reason. The co-infection rate in this study was higher than a study done at China [9], which might be due to the start of anti-TB chemotherapy before stool examination in Chinese study and the difference in the study setting.

Intestinal parasites and TB, either way, might be a risk factor one to the other [3]. TB patients harbour more intestinal parasites (protozoa and helminths) infections than TB free household contacts (COR = 2.85; 95% CI = 1.18–6.87) in this study, suggesting that individuals with parasite infections are more susceptible to get TB. Similarly this was reported by previous studies conducted in different settings [3, 11, 14]. However; there was no difference between persons with PTB and healthy controls in a study from rural China [10], which might be due to the start of anti-TB chemotherapy before stool examination. In our study, the rate of intestinal helminths is significantly higher among TB patients, (COR = 5.37; 95% CI = 1.14–25.25). The studies by Elias et al., in Gondar, Ethiopia [16] and Tristão- Sá et al.*,* in Brazil [14] support our finding. However, the study conducted in Gondar by Abate et al.*,* in Gondar, Ethiopia [7] and in Tanzania by Mhimbira et al.*,* in Tanzania [15] did not show statisticially significant difference between the TB patients and TB free household contacts. This might be due to the variability in the epidemiology of intestinal helminths in various geographic locations. Moreover, in the present study we used highly sensitive diagnostics; Xpert MTB/RIF Assay and MGIT 960 liquid culture, to rule out TB from the study controls. Multiple parasitic infection in TB patients was about 4 % in the present study. Similarly, mixed or more than one species of intestinal parasites co-infection among TB patients were reported by Alemu et al., in Arbaminch, Ethiopia [12], Alemayehu et al.*,* in Gondar, Ethiopia [11] and by Mhimbira et al., in Tanzania [15].

In this study; *G.lamblia*, *A. lumbricoides,*and *H.ana* were found with a significant difference in TB patients compared with controls. Likewise; a greater frequency of *G.lambia* infection in TB patients was reported by a study done at Gambo which is 250 km south of Addis Ababa, Ethiopia [30]. Similarly, *A.lumbricoides* was reported as the predominantly identified helminths in previous studies done in Ethiopia at Gondar [1, 3, 7] and Arbaminch [12]. Even though *H.nana* was significantly associated with TB in the present study, supportive findings are not found, which might be due to the absence of previous studies in similar settings. However, all the intestinal parasites identified in the present study except *C.parvum* was reported by different studies done in different settings and population [3, 5, 7, 10–13, 15]. The detection of *C.parvum* in TB patients in the present study might be due to the use of Modified Ziehl Neelsen staining method which was not included in the previous studies.

In the present study, intestinal parasitic infection on TB patients had a statistically significant association with BMI (AOR = 6.71; 95% CI = 1.65–27.25), which is supported by previous studies [10, 12, 15]. Even though, all two PTB patients from rural setting in this study were infected with intestinal parasites, it is difficult to compare with urban participants due to bigger numerical differences. However, studies from different parts of Ethiopia showed people who were living in rural areas were at risk of harboring intestinal parasites compared to urban dwellers [11, 12, 31]. Those who had dirty material in their finger nails were about nine times as likely to have intestinal parasites infection compared to those who did not have (AOR = 8.99; 95% CI = 2.46–32.78). This was supported by a study done by Abera et al.*,* in Tilili, Ethiopia [32]; where 47.9% of students who had dirty material in their finger nails were infected with helminthes infection. In the control group, there was no variable significantly associated with intestinal parasitic infection. Similarly; in a study done in China [10], annual labor time in farmlands more than 2 months was the only risk factor associated with overall infections in healthy controls. The limitations of this study were; only one-time stool specimen examined, Katho Katz technique not used to assess the parasitic load, nutritional assessments except BMI were not done and HIV antibody testing was not done for controls. Even though it is valid to assess BCG vaccination status by looking scars, it is sometimes hard to see. All these factors might have a potential bias and un assessed confounding.

## Conclusion

Although the study has limitations potentially leading to underdiagnosis due to one stool specimen is taken, the infection rate of intestinal parasites in TB patients and healthy household contacts had statistically significant difference. The most frequently identified intestinal parasites in TB patients with a statistical significant difference compared to controls were G*.lamblia, A. lumbricoides* and *H.nana*. Multiple parasitic infections were observed in TB patients but not in controls. Intestinal parasitic co-infection on TB patients had significant association with BMI and presence of dirty material in the participants’s finger nail. The consequence of co-infection on developing an active disease, disease severity and treatment efficacy needs to be investigated in future. A large-scale study with diverse population and wide geographical coverage should done.

## Abbreviations

AIDS: Acquired Immunodeficiency Syndrome
AOR: Adjusted Odds Ratio
BCG: Bacilli Calmete Gurine
BMI: Body Mass Index
CI: Confidence Interval
COR: Crude Odds Ratio
DOTs: Direct Observed Treatment short course
HIV: Human Immunodeficiency Virus
IP: Intestinal Parasites
LJ: Lowenstein Jenson
MGIT: Mycobacterium Growth Indicator Tube
MTB: *Mycobacterium tuberculosis*
NALC: N-Acetyl L-Cysteine
NaOH: Sodium Hydroxide
PTB: Pulmonary Tuberculosis
SPSS: Statistical Package for Social Sciences
TB: Tuberculosis
Th1: T helper type 1
WHO: World Health Organization
ZN: Ziehl Neelsen
